# Trends in suicide mortality in Montenegro from 2000 to 2018

**DOI:** 10.1186/s12991-021-00337-3

**Published:** 2021-02-22

**Authors:** Lidija Injac Stevović, Selman Repišti, Tamara Radojičić, Olivera Injac

**Affiliations:** 1 Faculty of Medicine, Clinical Centre of Montenegro, Psychiatric Clinic, Podgorica, Montenegro; 2 Department for Humanistic Study, University of Donja Gorica, Podgorica, Montenegro

**Keywords:** Suicide rates, Suicide methods, Montenegro, Well-being, Unemployment, Average salary

## Abstract

**Background:**

This is an ecological study that analyzes suicides committed in Montenegro during the 2000–2018 period, taking into account gender, age and methods of suicide.

**Methods:**

Suicide rates and trends up until 2009 were obtained from the official registers of Bureau of Statistics of Montenegro (MONSTAT) while the later data were obtained from the Department of Interior’s. MONSTAT also provided data on unemployment and average salary. As per statistical methods, descriptive and correlations were calculated.

**Results:**

The average crude suicide rate was 21.06, for males 29.93 and for females 12.42. Crude suicide rates were not associated with unemployment rate or average salary. However, the unemployment rate was significantly correlated with lethal methods of suicide, namely suicide by firearm and by hanging. Average net salary was negatively correlated with suicide by firearm.

**Conclusions:**

The ratio of males and females who committed suicide was 2.41. In the last three years, this ratio continues to rise in favor of males (reaching 4.29 in 2018). This could be explained by specific cultural features where males are expected to be the main financial contributors to the households. The labor market of Montenegro does not offer adequate opportunities to set and maintain a stable economic situation which puts additional pressure and stress on males.

## Introduction

Suicide could be regarded as one of the major public health concerns. It is defined as a deliberate and intentional act of ending one’s own life [8]. It can be differentiated from suicidal behavior which includes behaviors that do not necessarily end with death, such as deliberate self-harm, non-suicidal self injury, suicidal threats and suicidal gestures [2]. In 2016, around 800,000 suicides were committed worldwide, with an annual global age-standardized suicide rate of 10.53 per 100,000 [22]. Overall average crude suicide rate in Europe was 15.4 (24.7 in males and 6.6. in females), reported in the same year [22]. The crude suicide rate in upper-middle-income countries is approximately 7.5, with males to females ratio of 1.3 [21].

Montenegro belongs to the category of upper-middle-income countries, according to *The World Bank* (https://data.worldbank.org/income-level/upper-middle-income). It had around 622.400 inhabitants in 2018, according to estimates provided by MONSTAT. In this country, approximately 130 individuals per year die by suicide. For example, a crude suicide rate of 17.84 per 100,000 was reported for year 2016 with reduction of 1.85% up until 2018.

Various factors can influence suicide rates across countries. Most reported are socioeconomic factors such as high unemployment rates [16], unemployment risk and expectations of insufficient financial resources during unemployment [23]. It has been shown that economic crisis such as banking crises during the Great Depression also contributed to the increase in the number of committed suicides [6].

Population risk factors include (1) lack of social cohesion (fast changes in social structure, social isolation, and economic turmoil), (2) environmental factors (poor access to mental health care services, contents disseminated by the media, and access to lethal means), along with (3) three categories of individual factors (predisposing, mediating, and precipitating factors), as was outlined by Turecki and Brent [18]. These three types of individual factors encompass: genetics and early life adversity (predisposing factors); cognitive deficits, high levels of anxiety, impulsive aggression, chronic substance use (mediating factors), and acute substance use, adverse life events, sense of hopelessness, and behavioral disinhibition (precipitating factors).

In general, social well-being reflects on life satisfaction of human beings, and it is base for stabile and sustainable human development [19].^1^ Concept of the UNDP refers not only to economic insecurity, but also other sources of social inequalities, uncertainties and dissatisfactions with the quality of life, discrimination, injustice, distrust and more [19]. Also, some studies indicate that subjective well-being inequalities in the community could be addressed as relevant suicide risk factors [11], and concept of subjective well-being encompasses much broader aspects.

Gender plays a major role in attempting or committing suicide. Suicide is more frequently attempted by females unlike males who usually commit suicide more while choosing violent suicide methods [17]. For example, male–female ratios of committed suicide is more than 4.0 in most countries in the Balkans [21].

This study aims at determining overall and gender-specific crude suicide rates from 2000 to 2018 in Montenegro and their association with unemployment rate, average monthly net salaries, and two methods of committing suicide (firearms and hanging). The rationale of this study includes the fact that it is the first comprehensive study of suicide in Montenegro based on suicide data collected during extended period of time.

## Methods

Until 2009, the data on suicide mortality were collected and published by MONSTAT. From 2009 and on, the Department of Interiors gathers the data on suicide rates and trends and store them within their official registries. Procedure for collecting these data is as follows. In case of a violent death, the first records are provided by the doctor who arrives first to the investigation spot, usually from the Emergency Center. Further on, police examination takes place. Sociodemographic data about the victim are collected and interviews with family members are conducted in order to obtain information on possible causes of suicide. These data are further supported with those from medical documentation. Possible external causes of death are also under inquiry.

Montenegro suicidal data were collected for the last 19 years. The following variables were included in the statistical analysis: overall crude and gender-specific suicide rates, unemployment rates, average monthly net salaries, as well as committing suicide either by firearms or hanging.

### Data analysis

First, the average values and range of all the analysed variables were calculated. Next, Spearman’s coefficient of correlation was used to examine the direction and magnitude of relationships among variables.

## Results

Based on the data displayed in Table 1, the average suicide rate for the analyzed period was *M* = 21.06 (29.93 in males and 12.42 in females). The suicide ratio for the same period was 2.41 in favor of men. Furthermore, this ratio reached 4.29 in 2018. The highest overall suicide rate was 26.39 (in 2014) whereas the lowest one was 17.34 (in 2009). In males, the highest suicide rate was reported for year 2014 (42.00) and the lowest rate for year 2013 (22.49). In females, the highest reported suicide rate was 21.62 (in 2015) whereas the lowest one was 6.67 (in 2018).

**Table 1:** Overall and gender-specific crude suicide rates per 100,000 during the period of 2000–2018

Year	Total N of suicides	Male suicides	Female suicides	Total suicide rate	Suicide rate—males	Suicide rate—females
2000	112	78	34	18.57	26.26	11.11
2001	142	95	47	23.43	31.83	15.28
2002	120	84	36	19.72	28.03	11.66
2003	131	84	47	21.46	27.94	15.17
2004	119	93	26	19.44	30.88	8.36
2005	118	82	36	19.24	27.17	11.55
2006	138	107	31	22.51	35.42	9.97
2007	121	89	32	19.69	29.42	10.25
2008	126	80	46	20.47	26.39	14.73
2009	107	77	30	17.34	25.32	9.58
2010	159	108	51	25.69	35.38	16.25
2011	163	122	41	26.30	39.85	13.07
2012	148	100	48	23.86	32.64	15.29
2013	128	87	41	20.62	28.36	13.05
2014	164	129	35	26.39	42.00	11.13
2015	140	101	39	22.50	32.84	12.40
2016	111	83	28	17.84	26.97	8.9
2017	109	86	23	17.51	27.94	7.31
2018	109	88	21	17.51	28.60	6.67

Source: Department of Interiors and the MONSTAT

**Fig. 1:**
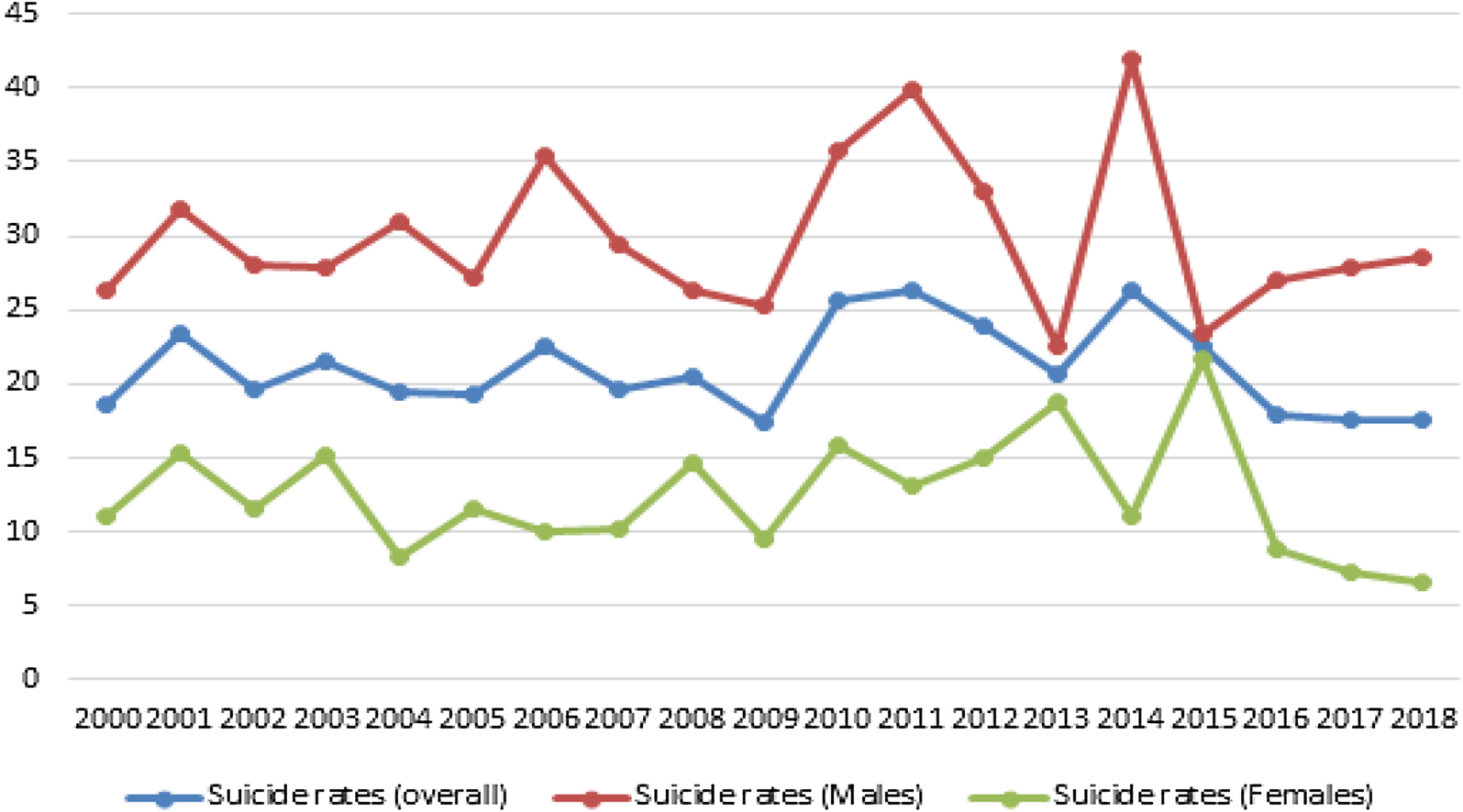
Overall and gender-specific suicide rates (per 100,000 population) from 2000 to 2018

Figure 1 shows trends in overall crude suicide rates as well as patterns of change in gender-specific suicide rates. From 2009 to 2015, there was a rapid increase and decrease in overall crude suicide rates, which peaked in 2014. In the last three years, this was an increasing trend in males unlike in females, where suicide rates decreased in this period of time.

As shown in Table 2, the unemployment rate ranged from 15.2 (in 2018) to 30.3 (in 2005). The average unemployment rate was 20.52. It should be noticed that the unemployment rates were very high in the period before Montenegrin independence referendum in 2006 (27.7 and 30.3 in 2004 and 2005) and in the year of the referendum (29.6). Next year, the unemployment rate fell to 19.4. With regard to monthly salaries, the lowest one was recorded in 2000 (97€) and the highest one was that of 2018 (511€). The average monthly salary for the analysed period was 360.05€. Average number of suicides committed by firearms was 45.24 (ranging from 30 in 2015 to 58 in 2011) and average number of suicides with the use of hanging was 59.24 (from 44 in 2017 to 72 in 2006).

**Table 2:** Unemployment rate, average salary, and number of suicides by firearms and hanging

Year	Unemployment rate (in %)	Average net salary (in euros)	Suicides by firearms (X72-X75)	Suicides by hanging (X70)
2000	19.2	97^a^	NA	NA
2001	21.2	108^a^	NA	NA
2002	20.7	149	56	50
2003	22.7	174	52	66
2004	27.7	195	57	53
2005	30.3	213	47	55
2006	29.6	282	50	72
2007	19.4	338	47	52
2008	16.8	416	47	64
2009	19.1	463	43	58
2010	19.7	479	45	66
2011	19.7	484	58	70
2012	19.7	487	51	63
2013	19.5	479	40	58
2014	18.0	477	35	72
2015	17.6	480	30	64
2016	17.7	499	32	51
2017	16.1	510	36	44
2018	15.2	511	43	49

Source: Department of Interiors and the MONSTAT

*NA* data not available

^a^In 2000 and 2001, salaries were paid in Deutsche Marks (DM); they were displayed in the table after conversion into euros (1 euro = 1.9558 DM)

As shown in Table 3, neither overall nor gender-specific crude suicide rates were associated with year, average net salary, or unemployment rate. Although statistically insignificant, the relationships of suicide rates with unemployment rates were positive and stronger than those of years and average net salary.

**Table 3:** Suicide rates correlated with unemployment rates and average salaries (Spearman's rank coefficients)

	Suicide rate		
	Overall	Males	Females
Year	− .067	− .025	− .175
Average net salary	− .051	.051	− .171
Unemployment rate	.337	.329	.237

As displayed in Table 4, the higher average net salary, the lower number of suicides by firearms (*r*_*s*_ = 0.544, *p* < 0.05); however, the relationship between average net salary and number of suicides by hanging was low and statistically insignificant. Unemployment rate correlated moderately with number of suicides by firearms (*r*_*s*_ = − 0.693, *p* < 0.01) and strongly with number of suicides by hanging (*r*_*s*_ = 0.896, *p* < 0.01).

**Table 4:** Spearman’s rank correlations between specific variables

	Suicide method	
	Firearms	Hanging
Average net salary	− .544*	− .194
Unemployment rate	.693**	.896**

^*^ p < .05; ** p < .01

## Discussion

The aim of the current study was to provide comprehensive overview of data on suicide rates and trends and their associated variables in Montenegro between 2000 and 2018. Results indicate that Montenegro belongs to the category of countries with high suicide rates (i.e. 20–24.9, e.g. according to [14], as this rate is around 21.06. In the last three years (2016–2018), suicide rates reached the point lower than the average rate calculated for that whole period of time, placing Montenegro in the category of countries with moderate suicide rates (i.e. 10–19.9, [14]. The overall trend in the last three years could be a result of the better quality of mental health services and the increased number of non-governmental organizations (NGOs) primarily focused on different aspects of mental health protection and implementation of projects in this domain. Crude suicide rates in females were much lower compared to those reported before 2016. Injac Stevovic and Vodopic [9] reported that most suicides attempts by females in Montenegro were associated with poisoning with psychotropic drugs, especially benzodiazepines. Up until 2015, these drugs have been available without a prescription which could explain reduced availability of benzodiazepine in subsequent years [15].

In comparison to its neighbour countries, Bosnia and Herzegovina and Serbia, Montenegro had higher crude suicide rates for the period 2007–2011 and 2006–2010, respectively. To be more specific, crude suicide rates in Bosnia and Herzegovina ranged from 12.3 in 2008 to 14.6 in 2007 [3] whereas in Serbia these rates ranged from 16.58 in 2010 to 19.43 in 2006 [4]. In this period, the lowest crude suicide rate in Montenegro was 17.34 in 2009 and the highest crude suicide rate was 25.69 in 2010.

Overall and gender-specific suicide rates were not in statistically significant relationships neither with year nor with average net salary. Hence, suicide rates did not increase steadily as time passes and this type of rates is not influenced by average net salary growth. It should be taken into account that only data for average salaries were available and not those for median salaries which could be considered as better indicator of individual income.

Nonsignificant correlation between overall suicide rates and unemployment was obtained. This finding was in accordance with findings obtained by Gutierrez-Barroso et al. [7]. In fact, as research evidence showed (e.g. [10], unstable employment is better predictor of suicide rates. However, some studies (e.g. [13] revealed that unemployment and lack of job security are important factors that can increase risk of suicide. Other studies (e.g. [1] underlined the possibility that mental health could mediate the relationship between unemployment and suicide rates. In spite of that, numbers of suicides by firearms and hanging were significantly associated with unemployment rate. Additionally, higher net salary seems to have negative relationship with suicide by firearms. In other words, unemployment and low salaries are linked to suicides committed by firearms.

These results should be interpreted with caution as unemployment data may not reflect accurately the labour situation in the country. Official employment and unemployment data do not include information on those who are not looking for a job anymore, those who have been deprived of working abilities, or those who are working in undocumented positions [23].

Overall suicide rates were relatively stable between 2000 and 2008 with minor oscillations. Notable change can be observed in data from 2009 where the suicide rate drops down below 17.5 and already in 2010 it increases above 25, reaching its highest point of more than 26 in 2014 (placing Montenegro among countries with very high suicide rates). This trend in suicide rates was highlighted by Fountoulakis et al. [5].

Males are 2.41 times more likely to commit suicide compared to females, which is in accordance to previously conducted studies on gender differences in commiting suicide [17], 20]. In 2018, this ratio was even 4.29. This finding can be explained with regards to Montenegrin cultural context which imposes high expectations on males in securing financial and material stability of households. Due to these expectations, it is plausible to assume that males in Montenegro are more prone to experience intense stress and pressure during times of economic, political and social disturbances, in comparison to females. Males’ reactions on economical changes related to employment and the average salary, seem to be more severe and lead to more extreme behavioral acts than those of females [12]. The risk of suicide in different population groups change during times of economic crisis or uncertainty. Men are more vulnerable than women to the adverse effects of economic recession, what also include suicide risk [11]:39).

Montenegro, as a small state, goes through the processes of transition (economic, political, social) in last three decades, what causes uncertain political and economic environment, and inevitably effects and concerns for well-being of citizens. By the sources reported from World Bank, poverty in Montenegro dropped about 11% in the mid-2000s to its minimum of 4.9% in 2008, and rising again to 8.6% in 2013, with a huge proportion of vulnerable categories, which are close to the poverty line [20]. Possibly, it indicates correlation between the suicide ratio and poverty, but also correlation with unstable socioeconomic situation in Montenegro.

The main advantages of the study are summarized as follows. Analysed data were obtained from the reliable and valid source and collected in a systematic way according to official Department of Interior’s procedures. In addition to this, they encompass an extensive time period from 2000 to 2018. Database used in the present study has potential to inform statistical registries on suicide rates and trends of worldwide organizations such as WHO.

Further research should focus on age-adjusted suicide rates in Montenegro and should analyse data for other methods of suicide (e.g. suffocation, drowning, poisoning, wrist cutting). At the same time, these recommendations can be considered as limitations of the present study. Another recommendation could be investigating the relationship of mental health, online and offline social networking, some other socioeconomic characteristics (e.g. marital status and educational level), and urban population growth with crude and age-adjusted suicide rates in Montenegro.

## Conclusion

To sum up, male–female suicide ratio increases over time in Montenegro, probably due to unstable socioeconomic situation. Based on crude suicide rates in the last three years, Montenegro belongs to countries with moderate risk for suicide. It seems that unemployment rate does not have significant impact on overall suicide rates; however, number of suicides committed by firearms and hanging was strongly associated with unemployment rates.

## Data Availability

All data are available.

## Abbreviations

MONSTAT: Statistical Office of Montenegro
WHO: World Health Organization
UNDP: United Nations Development Programme

## Footnotes

1 Human Development Report (1994) highlighted seven threats to human security: economic security, food security, health security, environmental security, personal security, community security and political security [18: p 24–25].

## References

[1] Agerbo E, Nordentoft M, Mortensen PB (2002). Familial, psychiatric, and socioeconomic risk factors for suicide in young people: nested case-control study. BMJ.

[2] Apter A (2010). Clinical aspects of suicidal behavior relevant to genetics. Eur Psychiatry.

[3] Bojanić N, Srdanović M (2012). Suicide rate in Bosnia and Herzegovina with special review on Federation of Bosnia and Herzegovina—Comparative analysis. Crim Just Issues J Crim Just Security.

[4] Dedić G (2014). Gender differences in suicide in Serbia within the period 2006–2010. Vojnosanitet Pregl.

[5] Fountoulakis KN, Kawohl W, Theodorakis PN, Kerkhof AJ, Navickas A, Höschl C, Lecic-Tosevski D, Sorel E, Rancans E, Palova E, Juckel G, Isacsson G, Jagodic HK, Botezat-Antonescu I, Warnke I, Rybakowski J, Azorin JM, Cookson J, Waddington J, Pregelj P, Demyttenaere K, Hranov LG, Stevovic LI, Pezawas L, Adida M, Figuera ML, Pompili M, Jakovljević M, Vichi M, Perugi G, Andreassen O, Vukovic O, Mavrogiorgou P, Varnik P, Bech P, Dome P, Winkler P, Salokangas RK, From T, Danileviciute V, Gonda X, Rihmer Z, Benhalima JF, Grady A, Leadholm AK, Soendergaard S, Nordt C, Lopez-Ibor J (2014). Relationship of suicide rates to economic variables in Europe: 2000–2011. Br J Psychiatry.

[6] Gunnell D, Platt S, Hawton K (2009). The economic crisis and suicide. BMJ.

[7] Gutierrez-Barroso J, Barragan-Medero F, Perez-Jorge D (2018). Suicide in Europe Countries: a multivariate approach analysis. Global J Health Sci.

[8] Gvion Y, Apter A (2012). Suicide and suicidal behavior. Public Health Rev.

[9] Injac Stevovic L, Vodopic S (2017). Attempted suicide in Podgorica, Montenegro: higher rates in females and unemployed males. Ann Gen Psychiatry.

[10] Kim C, Cho Y (2017). Does unstable employment have an association with suicide rates among the young?. Int J Environ Res Public Health.

[11] McDaid D. Socioeconomic disadvantage and suicidal behaviour during times of economic recession and recovery. In: Socioeconomic Disadvantage and Suicidal Behaviour, Samaritans Registered Office, Ewell, UK. 2017.

[12] Medojević D. Socio-demografske osobenosti suicidalne populacije u Crnoj Gori od 1997 do 2007. Sociološka Luča V/1. 2011.

[13] Nordt C, Warnke I, Seifritz E, Kawohl W (2015). Modeling suicide and uneployment: a longitudinal analysis covering 63 countries: 2000–11. Lancet Psychiatry.

[14] Peković M (2010). Psychiatry [Psihijatrija].

[15] Rule book on the form and content of prescriptions, criteria for classification of medicines, and the manner of prescribing and dispensing medicine (15/10/2015). file:///C:/Users/Lenovo/Downloads/Pravilnik%20o%20obrascu%20i%20sadr%C5%BEini%20recepta,%20kriterijumima%20za%20klasifikaciju%20ljekova,%20kao%20i%20na%C4%8Dinu%20propisivanja%20i%20izdavanja%20ljekova.pdf (09/06/20)

[16] Stuckler D, Basu S, Suhrcke M, Coutts A, McKee M (2009). The public health effect of economic crises and alternative policy responses in Europe: an empirical analysis. Lancet.

[17] Tsirigotis K, Gruszczynski W, Tsirigotis-Wołoszczak M (2010). Indirect (chronic) self-destructiveness and modes of suicide attempts. Archi Med Sci.

[18] Turecki G, Brent DA (2016). Suicide and suicidal behaviour. Lancet.

[19] United Nations Development Program (1994). Human development report.

[20] World Bank (2016) Partnership framework for Montenegro for period 2016–2020

[21] World Health Organization (2014). Preventing suicide: A global imperative.

[22] World Health Organization. Suicide Rates (per 100,000 Population). Global Health Observatory. Geneva: WHO; 2016

[23] Yuryev A, Varnik A, Varnik P, Sisask M, Leppik L (2010) Employment status influences suicide mortality in Europe. International Journal of Social Psychiatry 58(1):62–68. https://data.worldbank.org/income-level/upper-middle-income. Accessed 25 Dec 19. 10.1177/0020764010387059 21088037

